# The effect of Mediterranean diet on nutritional status, muscle mass and strength, and inflammatory factors in patients with colorectal cancer-induced cachexia: study protocol for a randomized clinical trial

**DOI:** 10.1186/s13063-022-06985-4

**Published:** 2022-12-14

**Authors:** Amir Bagheri, Mohammad Babaei, Saied Rezaei, Zoya Asl Motallebnejad, Maryam Ganjalikhani, Mahsa Malekahmadi, Ahmad Esmaillzadeh

**Affiliations:** 1 Department of Community Nutrition, School of Nutritional Sciences and Dietetics, Tehran University of Medical Sciences, Tehran, Iran; 2 Department of Radiation Oncology, Cancer Institute, Imam Khomeini Hospital Complex, Tehran University of Medical Sciences, Tehran, Iran; 3 Radiation Oncology Research Center, Cancer Institute, Tehran University of Medical Sciences, Tehran, Iran; 4 Imam Khomeini Hospital Complex, Tehran University of Medical Sciences, Tehran, Iran; 5 School of Medicine, Shahid Beheshti University of Medical Sciences, Tehran, Iran; 6 Department of Clinical Nutrition, School of Nutritional Sciences and Dietetics, Tehran University of Medical Sciences, Tehran, Iran; 7 Obesity and Eating Habits Research Center, Endocrinology and Metabolism Molecular - Cellular Sciences Institute, Tehran University of Medical Sciences, Tehran, Iran; 8 Department of Community Nutrition, School of Nutrition and Food Science, Isfahan University of Medical Sciences, Isfahan, Iran

**Keywords:** Cancer cachexia, Colorectal cancer, Mediterranean diet, Nutritional status, Inflammation, Muscle health

## Abstract

**Background:**

Current dietary strategies to manage cancer cachexia and the relevant outcomes did not provide a comprehensive solution. This study will evaluate the effect of a Mediterranean diet on inflammatory markers, nutritional status, muscle mass, and strength among patients with cancer cachexia (CC).

**Methods:**

This will be a randomized clinical trial involving men and women diagnosed with localized or advanced colorectal cancer-induced cachexia. In total, 40 patients with CC will be recruited based on inclusion criteria and then these patients will be randomly allocated to receive either a Mediterranean diet (*n* = 20) or only routine nutritional advice (*n* = 20) for 8 weeks. The primary outcome will be nutritional status, muscle mass and strength, and serum concentrations of inflammatory markers including interleukin-6 (IL-6), tumor necrosis factor-alpha (TNF-α), and high-sensitive C-reactive protein (hs-CRP). Moreover, we will consider serum albumin and total protein levels, complete blood count (CBC), and quality of life as the secondary outcomes. All outcomes will be measured at the beginning and end (the eighth week) of the study. We will assess participants’ adherence to the prescribed diets by using a 1-day food record in the second, fourth, sixth, and eighth weeks of the study.

**Discussion:**

Along with adequate calorie and protein intake in cancer cachexia, reducing inflammatory cytokines might be a useful strategy for maintaining nutritional status and body composition. Mediterranean diet has been shown to have anti-inflammatory properties, and by its components, it might help patients with cachexia to have a better nutritional status and quality of life.

**Trial registration:**

Iranian Registry of Clinical Trials (www.irct.ir) RCT20211027052884N1. Prospectively registered on November 09, 2021.

**Supplementary Information:**

The online version contains supplementary material available at 10.1186/s13063-022-06985-4.

## Background

Cancer cachexia (CC), defined as a multifactorial syndrome associated with continuous loss of skeletal muscle mass (with or without fat loss), accounts for 50% of all deaths in patients with cancer [1, 2]. Despite the prevalence of this condition among most cancer patients, 50% of patients with colorectal cancer are at risk to develop cachexia [3]. It is associated with reduced quality of life, increased length of hospital stays, increased healthcare costs, and elevated risk of mortality [1].

Systemic and acute inflammation plays a major role in the pathophysiology of CC [4]. Increased concentrations of inflammatory factors in these patients disrupt the function of leptin, ghrelin, neuropeptide Y, and the neuroendocrine axis, thereby continuously stimulating the anorexia pathways [4] and inducing hypercatabolic states, muscle destruction, and increased lipolysis [4]. On the other hand, chemotherapy and radiotherapy in these patients further exacerbate these conditions through increasing nausea, dysphagia, mucositis, and malabsorption [4]. Earlier studies have examined the effect of nutritional counseling on these patients and reached conflicting findings. While some investigators reported the efficacy of nutritional counseling on increasing energy and protein intakes in patients with CC, such diets did not result in significant changes in body weight, muscle mass, and strength [5, 6]. Mediterranean diet has been shown to effectively reduce inflammation [7, 8], cancer incidence, and mortality [9]. On the other hand, a significant positive relationship was seen between adherence to the Mediterranean diet and muscle mass and strength [10]. Consumption of the Mediterranean diet was also protectively associated with frailty and functional disability, as indicators of skeletal muscle function [11]. Despite the protective associations between the consumption of the Mediterranean diet and cancer and muscle health, we are aware of no clinical trial examining the effect of the Mediterranean diet on cancer cachexia. This dietary pattern is due to a high content of monounsaturated fatty acids (MUFA), polyunsaturated fatty acids (PUFA), oleuropein, hydroxytyrosol, polyphenols, B vitamins, antioxidant vitamins, and minerals which present in plant foods, fish, nuts, and extra virgin olive oil, which has been associated with anti-inflammatory properties through reducing arachidonic acid, inflammatory prostaglandins, and leukotrienes [12, 13]. Reducing inflammation through upregulating insulin and insulin-like growth factor-1 (IGF-1) which decrease protein degradation can elevate overall muscle health [14]. The current clinical trial will therefore be done to examine the effectiveness and superiority of the Mediterranean diet on nutritional status, muscle mass and strength, and inflammatory factors in patients with cancer-induced cachexia compared to routine nutritional care.

## Methods

### Participants

**Fig. 1:**
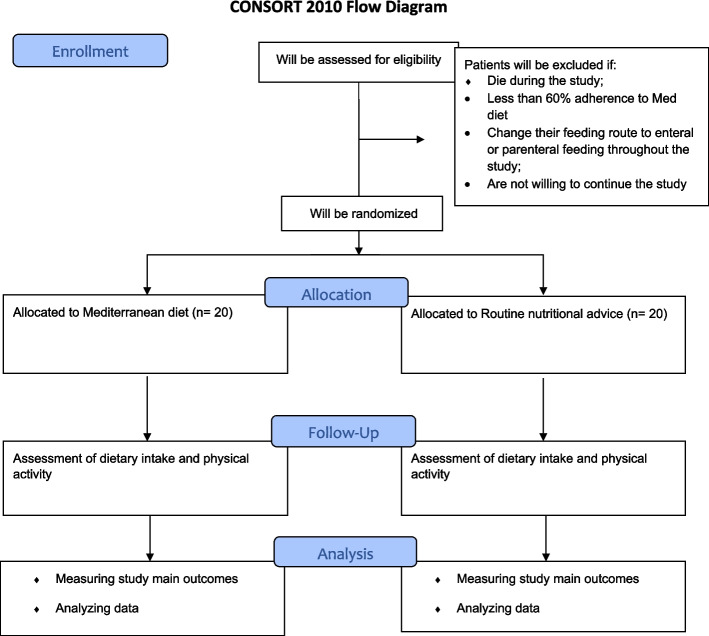
Flowchart of the study

This will be a randomized effectiveness controlled clinical trial among patients with localized or advanced colorectal cancers whose type of cancer will be confirmed by an oncologist through histopathology. Patients will be selected based on inclusion criteria from Imam Khomeini hospital, Tehran University of Medical Sciences in Tehran, Iran. Furthermore, the trial has been registered at www.irct.ir (IRCT20211027052884N1) on November 09, 2021. All items from the World Health Organization Trial Registration Data Set can be found within the current protocol that is registered in the Iranian Clinical Trials registry. The flow diagram of the study design is shown in Fig. 1.

### Inclusion criteria

In the study, we will recruit patients who have the following criteria: (1) patients with colorectal cancers in stages II–IV according to the TNM UICC 2010 method (based on the oncologist diagnosis) [15]; (2) patients with cachexia according to the Global Leadership Initiative on Malnutrition (GLIM) criteria [16]; (3) successful qualification to first-line radiotherapy or chemotherapy according to guideline [17]; (4) patients’ functional status is at least ≥70% according to Karnofsky scale [18]. The lower Karnofsky score indicates the lower chances of surviving; (5) individuals who have no contraindications for oral feeding and are able to be fed orally; (6) patients without acute uncontrolled underlying diseases such as kidney and/or liver failure; (7) patients at age 40 or older; and (8) men or women.

### Non-inclusion criteria

Patients with the following conditions will not be included in the study: (1) patients who need nutritional support (tube or intravenous feeding) and (2) patients with a history of allergy to any component of the Mediterranean diet, such as olive oil or nuts.

### Exclusion criteria

Patients who meet the following conditions will be excluded from the study and will not be included in the per-protocol analysis: (1) patients who die during the study; (2) patients with less than 60% adherence to the Mediterranean diet based on the prescribed energy and macronutrients; (3) individuals who change their eating route from oral feeding to enteral or parenteral feeding throughout the study; (4) patients that are not willing to continue the study for any reason; and (5) patients who change their treatment protocol during the study.

### Study design

After obtaining informed written consent, all required measurements will be performed at the beginning of the study. Then, stratified block randomization will be done based on the type of cancer (colon or rectum) and BMI (≤23, > 23) using the www.randomization.com website. The statistician will fold the paper containing a randomization number and put them in the envelopes and write the code on them. The envelopes will be kept at the enrollment center. Then, for each qualified patient, the nutritionist randomly picks one of the envelopes after shuffling and allocates patients into control and intervention groups. It is not possible to blind study participants or staff in this study due to nutritional interventions. Nevertheless, the outcome assessor will be blinded to the group assignment.

**Fig. 2:**
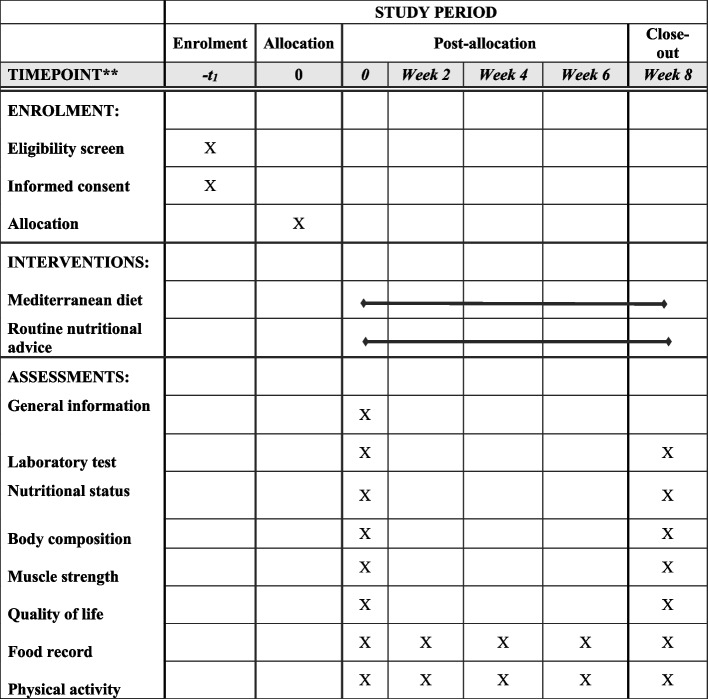
SPIRIT diagram of the recommended content for the schedule of enrolment, interventions, and assessments

Regarding colorectal cancer treatment, subjects with rectal cancer undergoing first-line radiotherapy and subjects with colon cancer undergoing first-line chemotherapy with capecitabine and oxaliplatin (CAPOX), 5-fluorouracil-leucovorin-irinotecan (FOLFIRI), 5-fluorouracil and oxaliplatin (FOLFOX), or capecitabine will be included [17]. As explained, people will be placed in separate blocks based on the type of cancer. The duration of the study for both intervention and control groups will be 8 weeks. Participants in the intervention group will receive a Mediterranean diet menu along with extra virgin olive oil, while participants in the control group will receive nutritional advice for cancer patients and dietary recommendations for weight gain. To assess participants’ adherence to the proposed diets, a 1-day food record will be used in the second, fourth, sixth, and eighth weeks of the study. Moreover, to encourage patients to follow the diet, all patients will be contacted weekly during the study. Participants will be asked to maintain their normal daily activities during the study. In addition, individuals’ physical activity will be monitored through completing a 1-day record in the second, fourth, sixth, and eighth weeks of the study. At the end of the study, all required measurements will be taken again in both groups. A Standard Protocol Items: Recommendations for Interventional Trials (SPIRIT) diagram for the trial schedule is indicated in Fig. 2.

### Intervention

The main intervention in this study is the administration of a Mediterranean diet. The energy required by patients in the intervention group will be computed according to the recommendations of the ASPEN guideline [19]. First, we will start with 25 kcal per kg of the current body weight and then gradually will move over 2 weeks to 35 kcal. Energy will be divided as follows between the macronutrients [20]: 35% of calories from fat (less than 7% saturated, 10–15% PUFA and the rest from MUFA), 45% of calories from carbohydrates, and 20% of calories from proteins. Then, by emphasizing the recommended food groups in the Mediterranean diet, the supply of these macronutrients from different food groups will be considered. Based on these food groups, a weekly dietary menu will be written for each patient. The Mediterranean diet will be designed based on the following recommendations: the use of extra virgin olive oil for cooking salads and other foods (participants will be given “extra virgin olive oil” free of charge), consumption of two or more exchanges per day of vegetables, three or more exchanges per day of fresh fruit, three or more exchanges per week of legumes, two or more exchanges per week of fish or seafoods, and three or more exchanges per week of nuts or seeds will be highlighted. The use of white meats instead of red or processed meats will also be focused. There will be also some recommendations to limit the consumption of butter, cream, fast foods, sweets, and sugary drinks. To increase the adherence to the dietary intervention, all the dietary menus will be personalized based on dietary habits of each person in the study.

In the control group, routine nutritional recommendations about weight gain and prevention of weight loss in cancer patients based on the clinical guidelines [21] will be given as brochures. All the points in these brochures will be explained to patients orally in a training session. As mentioned above, this group of patients, like the patients in the intervention group, will be followed up during the study period.

### Assessment of study outcomes

In the current study, the main primary outcome would be nutritional status, fat-free mass, muscle strength, and inflammatory markers including interleukin-6 (IL-6), tumor necrosis factor-alpha (TNF-α), and high-sensitive C-reactive protein (hs-CRP). Moreover, the following variables will be considered as the secondary outcome: serum albumin and total protein, complete blood count (CBC), and quality of life. All primary and secondary outcomes will be assessed at the beginning and end (the eighth week) of the study.

#### Anthropometric assessment

Height will be assessed using a tape measure in a standing position when the person without shoes and heels will close to the wall from the back, the knees will be straight and without bending, and the patient will be looking straight ahead and the eyes will level with the ears, and he will be asked to breathe slowly. Height was recorded with an accuracy of 0.1 cm. Handgrip strength, which is an indicator of muscle strength, will be measured using a digital dynamometer (Seahan, model SH5003, Seahan Co, South Korea). Body composition, including body fat percentage (PBF), body fat mass (BFM), fat-free mass (FFM), soft lean mass (SLM), body mass index (BMI), base metabolic rate (BMR), waist to hip ratio (WHR), and total body water (TBW), will be measured by the bio-electrical impedance analysis (BIA) (model: Portable Jawon medical). To get accurate results, the patient will be asked to be well hydrated, have not exercised in the past 6 h, and have not consumed caffeine, alcohol, or diuretics in the previous 24 h.

#### Nutritional status assessment

The nutritional status of individuals will be evaluated by the Patient Generated-Subjective Global Assessment Questionnaire (PG-SGA) [22]. This questionnaire assesses patients’ nutritional status based on information about a person’s medical history and diet history (weight change, food intake, gastrointestinal problems, and physical function) as well as clinical symptoms (body fat loss, muscle loss, and edema). According to the guidelines of the PG-SGA questionnaire, the severity of malnutrition will be defined in three following levels: scores between 0 and 1 indicate well-nourished status (A), scores between 2 and 8 indicate moderate or suspected malnutrition status (B), and scores of ≥ 9 indicate severe malnutrition status (C).

#### Biochemical assessment

In the fasting state, a 10-mL venous blood sample will be taken from each patient at the beginning and end of the trial. After separating the serum by centrifugation, the samples are stored at −80°C. Inflammatory factors (hs-CRP, IL-6, TNF-α) will be measured using the enzyme-linked immunosorbent assay (ELISA) by the use of commercially available kits. Serum albumin and total protein will be measured using an autoanalyzer (spectrophotometric method). The complete blood count (CBC) will also be done through a cell counter.

#### Assessment of other variables

General information about age, gender, education, economic status, medical history, medication and supplement use, and history of COVID-19 will be assessed by standard questionnaires. The quality of life of individuals will be assessed using the European Organization for Research and Treatment of Cancer quality of life questionnaire (EORTC QLQ-C30) (third edition) prepared by the European Agency for Research and Treatment of Cancer [23]. The validity and reliability of the questionnaires have already been evaluated in Iran [23]. In addition, the side effects of intervention as well as the treatment patients are taking such as diarrhea, nausea, constipation, vomiting, and appetite of patients will be assessed using the relevant items in this questionnaire. Also, the functional status of patients at the beginning and end of the study will also be assessed according to the Karnofsky scale. Participants will be asked to provide four 1-day food records throughout this trial. After providing the food records, the frequency of each food item will be converted to grams per day by considering the household measures of portion sizes. The daily energy and nutrient intake of each participant will be calculated by using Nutritionist IV software which is a modified food composition database based on the US Department of Agriculture.

### Data management

Special forms were designed by the researcher that were completed at any point in time and entered into the local database within a week of completion. Completed documentation is stored in a locked cabinet, with access to the principal investigator of the study. Forms are identified by a unique participant ID number and do not contain identifiable patient information. Data-driven queries are generated in the database daily, including date, range, and logic checks. Moreover, a list of all outcome data will be provided for patients who discontinue or deviate from study protocols. After collecting data, to ensure the data are complete and accurate, data will be double check by supervisors.

### Data monitoring

The Steering Committee of the university will oversee the project’s implementation and progress. The quality of the work, the patient admission process, and the data review will be carefully monitored by the University’s Executive Committee. If any changes are necessary for the continuation of the intervention or if the physician determines that the intervention should be stopped, the patient or his/her guardian will be informed of changes or discontinuation of the diet. Concerning the auditing plan, the progress and results of the study will be assessed by the University’s Steering Committee every 3 months. Moreover, the trial’s process and results will be independent of investigators and the sponsor.

### Sample size calculation

The sample size required for the current study was calculated using the formula (*n* = 2 [(*z*1−*α*/2 + z1−*β*)^2^ · *s*^2^]/*d*^2^), which has been proposed for parallel clinical trials [24]. Considering the nutritional status (score obtained from the PG-SGA questionnaire) as the key variable [25], type I error of 5% (*α* = 0.05), type II error of 20% (*β* = 0.20, study power = 80%), standard deviation of PG-SGA score (*S*= 4.1), and *d* as equal to 3.9 [25], the required sample size for each group will be 17 patients. However, to ensure a sufficient sample size at the end of the study and taking into account the 15% probable dropout, 20 patients in each group will be recruited according to the inclusion criteria. Considering that the samples will be given in this project are among the patients referred to the radio-oncology clinic and introduced by the radio-oncology specialist, therefore, while ensuring the correct selection of samples based on the inclusion criteria, the number of patients is sufficient to reach the desired sample size for this research project.

### Statistical analysis

Statistical analysis will be performed according to the intention-to-treat protocol; however, a per-protocol analysis will also be done. Thus, all randomized patients who have followed the Mediterranean diet for at least 2 weeks and who have the baseline measurements will be analyzed. With regard to missing data, the mean of each variable will be used to replace missing data. First, we will apply the Kolmogorov-Smirnov test to determine the normality of variables. In case of the non-normal distribution of a variable, a logarithmic transformation will be conducted. Independent sample’s *t*-test (or its non-parametric test Mann-Whitney *U*) will be used to compare the baseline values ​​of continuous variables between the two groups. Moreover, the chi-square test will be conducted to compare categorical variables. To examine the effect of the Mediterranean diet on the outcome variables, we will apply repeated measures ANOVA, in which the effect of time, intervention, and time*intervention interaction will be explored. In addition to the investigation of the crude means of variables in this analysis, we will compute adjusted means of outcome variables taking into account the baseline levels of the relevant variables. Changes in outcome variables will also be examined between the two groups using an analysis of covariance, in which the baseline values will be considered as covariates. Data will be analyzed using SPSS software version 26. *P*-value <0.05 will be considered as statistically significant.

## Discussion

Cachexia, as a prevalent complication in patients with cancer, has a significant influence on the length of hospital stay, healthcare costs, and quality of life and survival of patients with cancer. Despite the lack of consensus on the treatment of cancer cachexia, there is inconclusive data on the effect of a high-calorie and high-protein diet on cancer cachexia. Observational studies indicated that adherence to the Mediterranean diet might help in decreasing inflammation [7, 8], cancer incidence, and mortality [9]. Such a dietary pattern has also been shown to improve muscle strength (19, 20, 22) and can help in reducing the risk of sarcopenia [10, 11, 26]. In a clinical trial, consumption of a Mediterranean diet for 3 months among patients with lung cancer [27] resulted in improved “advanced lung cancer inflammation index,” which is computed as BMI×albumin/neutrophil-lymphocyte ratio, platelet count, and glycemic profile. Inflammation is one of the key features of cancer, due to the ability of tumor cells to recruit inflammatory cells and stimulate them to produce reactive oxygen spacious (ROS) [28, 29]. According to a large number of data, excessive oxidative stress in tissues and organs can cause inflammatory responses [30]. During tumor progression, inflammatory cytokines and chemokines can promote the survival and proliferation of cancer cells and tumor growth by stimulating angiogenesis [31]. As well as endogen factors, some exogenous factors such as an unbalanced diet and gut microbiota dysbiosis can cause inflammation in cancer [32]. The Mediterranean diet due to its high content of monounsaturated (MUFA) and polyunsaturated (PUFA) fatty acids has anti-inflammatory properties. Moreover, this dietary pattern is rich in polyphenols, B vitamins, antioxidant vitamins, and minerals [12]. There are some mechanisms to explain the anti-inflammatory effect of the Mediterranean diet: olive oil polyphenols (oleuropein, hydroxytyrosol) can reduce arachidonic acid and inflammatory prostaglandins and leukotrienes through inhibition of cellular enzymes such as phospholipase A2 (PLA2), cyclooxygenase (COX-1 and Cox-2), and lipoxygenase (LOX) [13]. These polyphenols also regulate the inflammatory response by suppressing the production of nitric oxide (NO) by inhibiting the expression of nitric oxide synthase (NOS) [30]. Furthermore, polyphenols are engaged in several steps of the activation process, making them an important pathway for the treatment and prevention of inflammatory diseases like cancer [33].

To the best of our knowledge, there is no further clinical trial examining the effect of the Mediterranean diet on nutritional status, body composition, inflammatory markers, and quality of life of patients with colorectal cancer-induced cachexia. Considering the point that muscle mass and strength, nutritional status, and quality of life of patients with cancer cachexia are continuously decreased and given the positive association of the Mediterranean diet with these factors in observational studies, we thought that this type of dietary pattern might be beneficial in improving the nutritional status of patients with colorectal cancer. The logic behind such hypothesis was the valuable health compounds of the Mediterranean diet including polyphenols, anti-oxidants, omega-3, and short-chain fatty acid as well as being a rich source of energy that may decrease inflammation and through which it may affect decreased muscle loss and improved nutritional status and quality of life.

### Strengths

This will be the first RCT on the effect of the Mediterranean diet on nutritional status, body composition, inflammatory factors, and quality of life among patients with colorectal cancer-induced cachexia. To increase adherence to the Mediterranean diet, the dietary menus will be tailored for each person based on their dietary habits. Moreover, we will give them extra virgin olive oil free of charge. Restricting the study participants to colorectal cancer-induced cachexia can be considered another strength of the study because different cancers might have different pathologies that can affect our findings. In addition, along with assessing the effect of the intervention on nutritional factors in cachexia, inflammatory markers will be evaluated to determine the intermediate mechanisms. Assessment of quality of life to identify if the Mediterranean diet has a beneficial effect on the quality of life of these patients is also of importance.

### Limitations

The most distinctive biomarker for examining adherence to the Mediterranean diet is the assessment of serum and urine hydroxytyrosol levels [34]. However, we will be unable to assess this biomarker as a measure of patients’ adherence to the Mediterranean diet due to limited funding. Therefore, we will use the average of four 24-h dietary records throughout the study to examine adherence to the prescribed diets. Although different modalities of cancer treatment may have an influence on nutritional status, we will stratify patients based on the type of treatment they receive. Moreover, it should be mentioned that blinding is impossible, and the risk of bias might be possible.

## Trial status

Enrollment of study participants has been started since Jan 2022. It is ongoing now.

## Supplementary Information

**Additional file 1.** Supplementary information.

## Data Availability

We will plan to publish all data in future studies. The raw data will be made available by request to the corresponding author.

## Abbreviations

CC: Cancer cachexia
MD: Mediterranean diet
IL-6: Interleukin-6
TNF-α: Tumor necrosis factor-alpha
hs-CRP: High-sensitive C-reactive protein
CBC: Complete blood count
ELISA: Enzyme-linked immunosorbent assay
PG-SGA: Patient Generated-Subjective Global Assessment Questionnaire
EORTC QLQ-C30: European Organization for Research and Treatment of Cancer Quality of Life Questionnaire
PUFA: Polyunsaturated fatty acids
MUFA: Monounsaturated fatty acids
BIA: Bio-electrical impedance analysis
PBF: Body fat percentage
BFM: Body fat mass
FFM: Fat-free mass
SLM: Soft lean mass
BMI: Body mass index
BMR: Base metabolic rate
WHR: Waist to hip ratio
