# Supplementary material for: The effect of Mediterranean diet on nutritional status, muscle mass and strength, and inflammatory factors in patients with colorectal cancer-induced cachexia: study protocol for a randomized clinical trial
Source: Trials. 2022 Dec 14;23:1015. doi: 10.1186/s13063-022-06985-4 (PMC9750717; doi:10.1186/s13063-022-06985-4)
Supplement: Supplementary file 1 — Additional file 1. Supplementary information. [file 13063_2022_6985_MOESM1_ESM.docx]

**Supplementary information**

Informed consent form patients to participate in the current research project

Dear Ms./Mr

You are hereby invited to participate in the aforementioned research. The information related to this research is presented in this service sheet and you are free to participate or not participate in this research. You don't have to make an immediate decision and you can ask your questions to the research team and consult with anyone you want to make a decision. Before signing this consent form, make sure that you have understood all the information in this form and that all your questions have been answered.

Conductor of the research

Ahmad Esmaillzadeh

1. I know that the goals of this research are:

"Effect of the Mediterranean diet on nutritional status, muscle mass and strength and inflammatory factors in patients with cachexia caused by colorectal cancer: a randomized controlled clinical trial"

2. I know that my participation in this research is completely voluntary and I am not forced to participate in this research. I was assured that if I am not willing to participate in this research, I will not be deprived of the usual diagnostic and therapeutic care, and my therapeutic relationship with the treatment center and my doctor will not be disturbed.

3. I know that even after agreeing to participate in the research, I can withdraw from the research whenever I want, after notifying the administrator, and my withdrawal from the research will not cause me to be deprived of receiving the usual medical services.

4. The way I cooperate in this research is that at the beginning of entering this project, my height, weight, and body composition, including muscle mass, body fat, and muscle strength will be checked. Then I will be asked questionnaires related to general information and medical history, food intake, and quality of life. It will take me half an hour to 45 minutes to fill out these questionnaires. In addition, the level of inflammatory factors (hs-CRP, IL-6, TNF-α), albumin, total protein, and CBC will be checked through blood tests. It is necessary to mention that all measurements will be done again at the end of the study (eighth week). I will not be responsible for any travel and blood testing costs.

5. The possible benefits of my participation in this study are as follows:

1) By cooperating with this plan, I can receive a free consultation to control or gain weight.

2) Information about your health status (muscle mass, muscle strength) and the level of inflammatory factors (hs-CRP, IL-6, TNF-α), albumin, total protein, and CBC

6. Possible damages and complications of participating in this research are as follows:

• This study will not have any adverse effects or consequences for the participants.

7. If I do not wish to participate in the research, the usual services (treatment, diagnosis, etc.) will be provided to me, the benefits and side effects of which are as follows:

• Free consultations for weight control.

8. I know that the people involved in this research have kept all the information related to me confidential and they are only allowed to publish the general and group results of this research without mentioning my name and details.

9. I know that the research ethics committee can have access to my information to monitor the observance of my rights.

10. I know that I will not be responsible for any of the research intervention costs as follows:

1) The cost of body composition measurement and inflammatory factor level tests (hs-CRP, IL-6, TNF-α), albumin, total protein, and CBC (twice)

2) Travel expenses

11. Dr. Amir Bagheri was introduced to me to answer and I was told to share with him and ask for guidance whenever there is a problem or question related to participating in the mentioned research.

His address and landline and cell phone numbers were presented to me as follows:

Address: Keshavarz Blvd., Naderi St., Hojat Dost Alley, Faculty of Nutrition and Dietetics, Tehran University of Medical Sciences

Mobile phone: ….

12. I know that if during and after the research, any problem, whether physical or mental, occurs to me due to participating in this research, the treatment of complications, its costs, and the related compensation will be the responsibility of the administrator.

13. I know that if I have problems or objections to the participants or the research process, I can contact the research ethics committee.

Tehran University of Medical Sciences address: Tehran, the intersection of Keshavarz Blvd and Qods St., Tehran University of Medical Sciences Headquarters Building, 5th floor, room 105 and raise your problem verbally or in writing.

14. This information and informed consent form is prepared in two copies and after signing, one copy will be at my disposal and the other copy will be at the manager's disposal.

I have read and understood the above-mentioned items and based on that, I declare my informed consent to participate in this research.

Participant's signature

I, Dr. Ahmad Esmaillzadeh, consider myself bound to implement the obligations related to the executive in the above provisions and undertake to ensure the rights and safety of the participants in this research.

Seal and signature of the researcher

Dr. Ahmed Ismailzadeh
